# Loss‐of‐function variant in *TDRD6* cause male infertility with severe oligo‐astheno‐teratozoospermia in human and mice

**DOI:** 10.1111/jcmm.18580

**Published:** 2024-09-27

**Authors:** Xinying Bi, Huijuan Jin, Feng Wan, Yanqing Xia, Haibin Guo, Suren Chen, Binbin Wang

**Affiliations:** ^1^ Center for Genetics National Research Institute for Family Planning Beijing China; ^2^ Graduate School, Chinese Academy of Medical Sciences & Peking Union Medical College Beijing China; ^3^ Key Laboratory of Cell Proliferation and Regulation Biology, Department of Biology, Ministry of Education, College of Life Sciences Beijing Normal University Beijing China; ^4^ The Reproductive Medicine Center, Henan Provincial People's Hospital People's Hospital of Zhengzhou University Zhengzhou Henan China

**Keywords:** chromatoid body, loss‐of‐function variant, male infertility, mutant mice, oligo‐astheno‐teratozoospermia, TDRD6

## Abstract

Oligo‐astheno‐teratozoospermia (OAT) is a common cause of male infertility, but the genetic basis of most OAT cases is still unknown. Here, one homozygous loss‐of‐function (LOF) variant in *TDRD6*, c.G1825T/p.Gly609X, was identified in an infertile patient with severe OAT by whole‐exome sequencing (WES) and Sanger confirmation. Furthermore, *Tdrd6*‐mutant mice (p.Gly615X; equivalent to p.Gly609X in human *TDRD6*) were generated. Remarkably, the *Tdrd6*‐mutated mice mimicked the severe OAT symptoms of the patient. In addition, the architecture of chromatoid bodies (CBs) were disrupted in round spermatids from *Tdrd6*‐mutant mice, leading to blocked spermatogenesis in the round spermatids. The assembly of PIWIL1, TDRD1, TDRD7 and DDX25 in CBs was disturbed in the *Tdrd6*‐mutant mice. Applying immunoprecipitation‐mass spectrometry (IP‐MS), we identified some TDRD6‐interacting partners, including CB proteins TDRD7, MAEL and PCBP1. Moreover, we described the assisted reproductive technology (ART) outcomes of the infertile patient and his partner. Altogether, our findings provide necessary evidences to support the idea that the homozygous LOF variant in *TDRD6* induces male infertility with severe OAT, suggesting that *TDRD6* could be a useful genetic diagnostic target for male infertility.

## 1 INTRODUCTION

Infertility affects approximately 15% of regularly sexually active couples, and has emerged as a serious public health issue.^1^ Males account for approximately half of all infertility cases, and the potential factors for male infertility are complex and diverse.^2^ Oligo‐astheno‐teratozoospermia (OAT) is a common cause of male infertility, which is characterized by a low number of spermatozoa with poor motility and morphological abnormalities.^3^ Many OAT cases have been diagnosed as idiopathic and are considered to be caused by genetic defects.^4^ With the widespread application of high‐throughput sequencing technologies, such as whole‐exome sequencing (WES), some OAT‐related genes have been identified in human cases of OAT, including *NANOS1*,^5^ *KATNAL2*,^4^ *KLHL10*,^6^ *MNS1* ^7^ and *CCDC34*.^8^ Although genetic defects that affect spermatogenesis are known to cause OAT symptoms, the aetiology of most infertile patients with OAT remains largely unclear and further studies are needed to reveal its pathogenesis.

In mammals, spermatogenesis is a tightly regulated differentiation process that involves complex events of genomic and epigenetic regulation.^9^ However, gene transcription during spermatogenesis is not continuous, rather it gradually decreases and comes to a complete halt as the nucleus condenses. Therefore, many mRNAs are presynthesized and stored at specific cytoplasmic sites, such as ribonucleoprotein granules called chromatoid bodies (CBs), for translation and function at specific developmental stages.^10^ CBs are unique cloud‐like structures in the cytoplasm from late pachytene spermatocytes to round spermatids and gradually disappear in elongated spermatids.^11^ CBs are relatively large ribonucleoprotein particles that mainly contain Piwi‐interacting RNAs (piRNAs), small RNAs, mRNAs, long noncoding RNAs and RNA‐binding proteins, which play key roles in chromatin compaction during sperm elongation.^12^ In addition to piRNA‐binding PIWI proteins (e.g. MIWI and MILI), tudor domain‐containing (TDRD) proteins, such as TDRD1, TDRD3, TDRD4, TDRD6 and TDRD7,^13^ are crucial components of CBs. Clinical and animal evidences have highlighted the essential role of TDRD family members in male fertility.^14^, ^15^, ^16^, ^17^, ^18^, ^19^ Loss‐of‐function (LOF) *TDRDs* variants^14^ have been identified in infertile men with OAT or nonobstructive azoospermia (NOA).

In this study, we identified one homozygous LOF variant (c.G1825T/p.Gly609X) in *TDRD6* in one infertile patient with severe OAT by WES. We further generated *Tdrd6*‐mutant mice (p.Gly615X; equivalent to the p.Gly609X variant in the patient) that generated no TDRD6 protein and perfectly mimicked the OAT phenotype. Moreover, we revealed that the assembly of PIWIL1, TDRD1, TDRD7 and DDX25 in CBs was disrupted in the *Tdrd6*‐mutant mice. By applying immunoprecipitation‐mass spectrometry (IP‐MS) and co‐immunoprecipitation (co‐IP), we further showed that TDRD6 interacted with TDRD7, MAEL and PCBP1 in CBs. Our findings provide essential evidences to support the idea that *TDRD6* is a solid OAT‐associated gene that is critical for CB architecture and transition from round spermatids to sperm.

## 2 MATERIALS AND METHODS

### 2.1 Study participants

We recruited approximately 110 patients with primary male infertility for this study. The proband was diagnosed with idiopathic OAT, had a normal karyotype (46, XY), and no microdeletions were found on the Y chromosome. Clinical examination showed that the patient had normal development of male external genitalia, normal bilateral testicular size and normal andrological hormone levels. No abnormalities were observed in the bilateral spermatic veins upon palpation. Written informed consent was obtained from all participants. This study was approved by the Ethics Committees of National Research Institute for Family Planning.

### 2.2 Semen analysis

Sperm counts were determined using a fertility counting chamber (Makler, Israel) under a light microscope. Sperm mobility was assessed via the application of a computer‐assisted sperm analysis system (SAS Medical, China). The sperm suspension was mounted on a glass slide, air‐dried and fixed with 4% PFA for 30 min at room temperature. The slides were stained with Papanicolaou solution (Solarbio, Beijing, China) and observed using a DM500 optical microscope (Leica, Germany).

### 2.3 WES and Sanger sequencing

Genomic DNA was extracted from the peripheral blood of the participants using a QIAamp DNA Blood Mini Kit (QIAGEN, USA). The Agilent SureSelect Human All Exon V6 Kit (Agilent Technologies, USA) was applied for exon capture, and the Illumina HiSeq X system was utilized to perform sequencing according to the manufacturer's instructions. The average sequencing depth on targets was ~100×. Reads were mapped to the human genome reference (GRCh37/hg19) by Burrows Wheeler Aligner software.^20^ ANNOVAR software^21^ was utilized for functional annotation via various databases, including dbSNP, 1000 Genomes Project, ExAC and HGMD. The Genome Analysis Toolkit (GATK 3.7)^22^ was employed to identify and quality filter the variants. Candidate genes were filtered according to the following criteria: missense, nonsense, frameshift or splicing site variants; variant frequency <1% from East Asian and the total population in the gnomAD v2.1.1 database (http://gnomad.broadinstitute.org). Sanger sequencing was applied to verify the variant detected by WES in the patient and his parents. The primers used for Sanger sequencing are listed in Table S1.

### 2.4 Expression plasmids and transient transfection

Human wild‐type (WT) *TDRD6* and *TDRD6* c.G1825T cDNAs were synthesized chemically (Sangon Biotech) and inserted into Flag‐tagged pCMV vectors (GenScript, China). Construction of expression plasmids in this study was confirmed by sequencing (Sango Biotech). HEK293T cells (ATCC, VA, USA) were cultured at 37°C in a 5% CO_2_ incubator with Dulbecco's modified Eagle's medium with 10% fetal bovine serum and 1% penicillin–streptomycin (HyClone, UT, USA). Transient transfection of HEK293T cells was performed using Lipofectamine 3000 transfection reagent (Thermo Fisher Scientific, CA, USA) following the manufacturer's protocol. Cells were harvested 48 h after transfection. The cycloheximide (CHX) used for the protein degradation analysis was purchased from MedChemExpress (MCE, Shanghai, China).

### 2.5 Mouse model

Animal experiments were approved by the Animal Care and Use Committee of the College of Life Sciences, Beijing Normal University. The p.Gly615X (GGG–TGA) variant that we created in mouse *Tdrd6* is equivalent to the p.Gly609X (GGA–TGA) variant in human *TDRD6*. Mouse *Tdrd6* (ENSMUSG00000040140) is located on mouse chromosome 17. Five exons were identified and the p.Gly615X variant was located on exon 1. Mouse zygotes were co‐injected with an RNA mixture of Cas9 mRNA (TriLink BioTechnologies, CA, USA), sgRNA and donor oligo. The injected zygotes were transferred into pseudopregnant recipients to obtain the F0 generation. DNA was extracted from tail tissue of 7‐day‐old offspring, and PCR amplification was carried out with genotyping primers using a Mouse Tissue Direct PCR Kit (Tiangen Biotech, China). A stable F1 generation (heterozygous mice) was obtained by mating positive F0 generation mice with WT C57BL/6J mice. Sequences of sgRNA, donor oligo and primers for genotyping are provided in Figure S1.

### 2.6 Fertility testing

To confirm the fertility of *Tdrd6*‐mutated male mice, natural mating tests were conducted. Briefly, adult *Tdrd6*‐mutated male mice and their littermate WT mice (*n* = 3 each) were mated with WT C57BL/6J females (male: female = 1:2) for 2 months. Mice were examined for vaginal plugs every morning. Female mice with vaginal plugs were separately fed, and female mice were replenished. The number of pregnant females and the number of pups per litter were recorded.

### 2.7 Generation of TDRD6 antibody

The N‐terminus of mouse TDRD6 was cloned into the pET‐N‐His‐C‐His vector and then transfected into the *Escherichia coli* ER2566 strain. Protein expression was induced by 1 mM IPTG at 32°C overnight. After centrifugation, the bacterial pellet was resuspended in buffer (50 mM Tris–HCl pH 8.0, 200 mM NaCl) and the proteins were released by sonication. After centrifugation, anti‐His beads were added to the supernatant and incubated overnight at 4°C. After washing, the recombinant protein was eluted with 250 mM imidazole. Recombinant TDRD6 protein was emulsified at a 1:1 ratio (*v*/*v*) with Freund's complete adjuvant and administered subcutaneously into female ICR mice (Charles River, Beijing, China) at multiple points. For two subsequent immunizations, the recombinant TDRD6 protein was emulsified with incomplete Freund's adjuvant at an interval of 2 weeks. One week after the last immunization, blood was collected, and the serum was separated. All reagents were purchased from Beyotime (Shanghai, China).

### 2.8 Histological analysis

The testes and caudal epididymis were dissected and fixed in 4% PFA overnight at 4°C. Fixed tissues were embedded in paraffin, sectioned (5‐μm thick), dewaxed and rehydrated. The sections were stained with haematoxylin–eosin (Solarbio, Beijing, China) before imaging using a Leica DM‐500 optical microscope (Leica Microsystems, German).

### 2.9 Immunofluorescence

Five‐micron paraffin‐embedded mouse testis sections were deparaffinized, rehydrated and subjected to antigen retrieval in 10 mM sodium citrate buffer. After permeabilization with 1% Triton X‐100 for 1 h, testis sections were blocked with 5% goat serum for 1 h. Mouse TDRD6 antiserum (homemade, 1:200) and rabbit anti‐DDX4 (Abcam, ab13840, 1:200) were added to the slide and incubated overnight at 4°C. After washing three times with 1 × PBS, the slides were incubated with Alexa Fluor 484‐labelled donkey anti‐mouse IgG and 555‐labelled donkey anti‐rabbit IgG (Beyotime, Shanghai, China, 1:200) for 1 h at room temperature. The nuclei were counterstained with DAPI dye (Beyotime, 1:500).

### 2.10 Western blotting

Proteins from mouse testes were extracted using RIPA lysis buffer containing 1 mM PMSF and protease inhibitors on ice. Supernatants were collected following centrifugation at 12,000 × g for 10 min. Proteins were electrophoresed in 10% SDS–PAGE gels and transferred to nitrocellulose membranes (GE Healthcare, USA). The blots were blocked in 5% milk and incubated with mouse TDRD6 antiserum (homemade, 1:1000) or rabbit anti‐DDX4 (Abcam, ab13840, 1:1000) overnight at 4°C, followed by incubation with anti‐rabbit or mouse IgG H&L (HRP) (Abmart, China, 1:10,000) for 1 h. The signals were evaluated using the Super ECL Plus Western Blotting Substrate and Tanon imaging system (China). All reagents were purchased from Applygen (Beijing, China).

### 2.11 Transmission electron microscopy

Mouse testes were fixed with 2.5% glutaraldehyde in 0.1 M phosphate buffer (PB) (pH 7.4) at 4°C. The samples were washed four times in PB and immersed in 1% OsO_4_ and 1.5% potassium ferricyanide aqueous solution at 4°C for 2 h. After washing, the samples were dehydrated through graded alcohol into pure acetone. Samples were infiltrated in a graded mixture of acetone and SPI‐PON812 resin, then the pure resin was changed. The specimens were embedded in pure resin with 1.5% BDMA, polymerized for 12 h at 45°C and 48 h at 60°C, cut into ultrathin sections (70‐nm thick), then stained with uranyl acetate and lead citrate for subsequent observation and photography with a Tecnai G2 Spirit 120 kV (FEI) electron microscope. All reagents were purchased from Zhongjingkeyi Technology (Beijing, China).

### 2.12 IP‐MS

Testis tissues were collected from 28‐day‐old *Tdrd6* ^M/M^ mice and their littermate controls (*n* = 3 in each group) and lysed with Pierce™ IP Lysis Buffer (Thermo Fisher, CA, USA). Protein lysates were incubated overnight with 2 μg TDRD6 antiserum at 4°C. Then, the lysates were incubated with 20 μL Pierce™ Protein A/G‐conjugated Agarose for 4 h at 4°C. IP products were digested by trypsin digestion for 4 h at 37°C. Separation was performed by Thermo UltiMate 3000 UHPLC (Thermo Scientific, MA, USA). The peptides that were separated by liquid phase chromatography were ionized by a nanoESI source and then passed to a tandem mass spectrometer Q‐Exactive HF‐X (Thermo Fisher Scientific, San Jose, CA) for data dependent acquisition mode detection. Protein identification uses experimental MS/MS data and aligns them with theoretical MS/MS data from the UniProt database to obtain the results. Mascot 2.3.02 search, quality control and iBAQ quantification were subsequently performed. The mass spectrometry proteomics data have been deposited to the ProteomeXchange Consortium with the dataset identifier PXD042162.

### 2.13 Co‐immunoprecipitation

Immunoprecipitation of TDRD6 from mouse testis protein lysates was performed as described above. Anti‐TDRD7 polyclonal antibody (Thermo Fisher, PA5‐143964, 1:1000), anti‐MAEL polyclonal antibody (Proteintech, 26666‐1‐AP, 1:1000) or anti‐PCBP1 polyclonal antibody (Proteintech, 14523‐1‐AP, 1:1000) was used for subsequent Western blotting detection.

### 2.14 CB isolation

CBs were isolated as described previously^23^ with some modifications. Germ cells were isolated from WT and *Tdrd6* ^M/M^ adult mice with 0.05% collagenase and filtered through a 100 μm cell strainer. The cells were fixed in 1% PFA solution for 20 min at room temperature. The fixed cells were lysed by sonication in RIPA lysis buffer containing 1 mM PMSF and protease inhibitors. The lysate was centrifuged at 300 × g for 10 min and the CB‐enriched pellet was resuspended in RIPA buffer. The CBs were immunoprecipitated using Dynabead Protein G (Invitrogen) coupled to rabbit polyclonal anti‐DDX4 (Abcam, ab13840) overnight at 4°C. Dynabeads were washed three times with RIPA buffer and the crosslinks of the isolated CBs were reversed by incubation at 70°C for 30 min. Anti‐PIWIL1 polyclonal antibody (Proteintech, 5659‐1‐AP, 1:1000), anti‐TDRD1 polyclonal antibody (Invitrogen, PA5‐88834, 1:1000), anti‐TDRD7 (Thermo Fisher, PA5‐143964, 1:1000), anti‐DDX25 polyclonal antibody (FineTest, FNab02302, Wuhan, China, 1:1000) or anti‐HNRNPA3 polyclonal antibody (Proteintech, 25142‐1‐AP, 1:1000) was used for subsequent Western blotting detection.

### 2.15 In silico analysis and molecular modelling

The pathogenicity of the variant was predicted by in silico analysis using MutationTaster (http://www.mutationtaster.org) and CADD (https://cadd.gs.washington.edu/). CLC Sequence Viewer 8 software was used to analyse the conservation of mutation sites in different species. The structures of the WT and mutant TDRD6 proteins were generated using SWISS‐MODEL. PyMol software (http://www.pymol.org) was used to visualize the 3D structures.

### 2.16 Statistics

Data were compared for statistical significance using GraphPad Prism version 5.01 (GraphPad Software). Unpaired, two‐tailed Student's *t*‐test was used for the statistical analyses. The data are presented as the mean ± SEM, and statistically significant differences are represented as *p < 0.05, **p < 0.01 and ***p < 0.001.

## 3 RESULTS

### 3.1 Identification of a homozygous LOF *TDRD6* variant in an infertile patient with severe OAT

**FIGURE 1:**
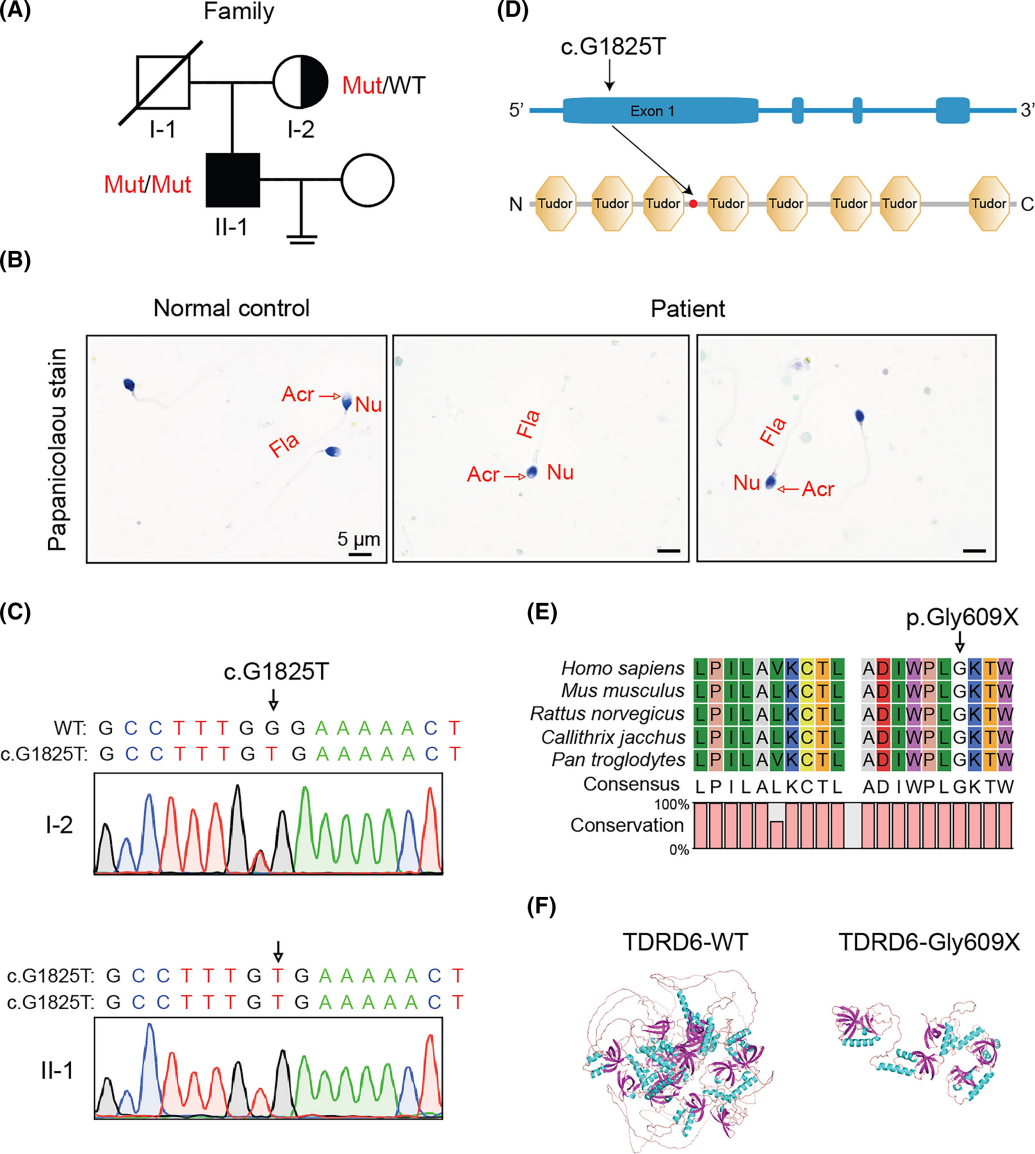
Identification of a LOF *TDRD6* variant in an infertile patient with severe oligo‐astheno‐teratozoospermia. (A) Pedigree analysis of the patient with the *TDRD6* variant. Black‐filled squares indicate infertile man in the family. (B) Papanicolaou staining was performed to reveal the morphology of sperm. (C) Sanger sequencing validated the homozygous *TDRD6* variant c.G1825T/p.Gly609X in the patient. (D) Schematic representation of the *TDRD6* exon structure (top) and predicted TDRD6 protein regions (bottom). Localization of the variant is indicated by the black arrows. (E) Conservation analysis of the affected amino acids among different species. (F) Predicted structure of the p.Gly609X variant.

An infertile man from a non‐consanguineous family was investigated in this study (Figure 1A). We analysed the semen parameters of the patient, and found a sharply reduced sperm count and no progressive mobile sperm (Table 1). The very few remaining sperm in patient were morphologically abnormal, as shown by Papanicolaou staining (Figure 1B).

**TABLE 1:** Semen characteristics and sperm morphology in the proband harbouring *TDRD6* variant.

	II‐1	Reference limits
Semen volume (mL)	3.9	1.5
Concentration (10^6^/mL)	4.3	15.0
Motility (%)	8.0	40.0
Progressive (%)	6.0	32.0
Normal morphology (%)	1.0	4.0

*Note*: Reference limits according to the WHO standards.

The proband had a normal karyotype (46, XY), and no microdeletions were found on the Y chromosome. WES was performed to explore the underlying genetic cause of the patient's severe OAT. Twenty potential pathogenic genes were screened out following the criteria: (1) variants affected coding exons or splice sites (including missense, nonsense, frameshift and splicing site variants); (2) minor allele frequency was <1% in gnomAD for autosomal variants and <1‰ for variants on the X chromosome; and (3) variants were not predicted as benign or likely benign based on the ACMG recommendations. All candidate pathogenic genes in the family were listed in Table S2.

Based on a comprehensive literature review of human disease symptoms and gene‐edited mouse phenotypes, we found that the testis‐specific gene *TDRD6* was the gene that was most significantly associated with male infertility, especially OAT, among all the screened genes. The c.G1825T/p.Gly609X variant in *TDRD6* has not yet been reported in the ExAC, 1000 genome and gnomAD databases (Table 2). Sanger sequencing identified the patient's mother (I‐2) as a heterozygous carrier of this variant, suggesting a recessive inheritance pattern (Figure 1C).

**TABLE 2:** Homozygous LOF *TDRD6* variant identified in one OAT‐affected man.

	Mut
cDNA alternation	c.G1825T
Protein alteration	p.Gly609X
Variant allele	Homozygous
Variant type	Nonsense
Allele frequency in human population	
All individuals in gnomAD	0
East Asians in gnomAD	0
Function prediction	
MutationTaster	Damaging
CADD	36

*TDRD6* is located in the p12.3 region of human chromosome 6. According to the Human Protein Atlas, human *TDRD6* mRNA is annotated as specifically expressed in testis tissue; within testes, *TDRD6* mRNA was restricted to the early spermatid subpopulation (Figure S2). The c.G1825T/p.Gly609X variant in *TDRD6* was predicted to generate a truncated TDRD6 lacking 4–8 tudor domains (Figure 1D and Figure S3A). Moreover, the amino acids affected by the variant were highly conserved during evolution (Figure 1E). To visualize the effect of p.Gly609X on the related protein, we constructed a three‐dimensional (3D)‐structural figure of TDRD6 using SWISS‐MODEL. The results show that many amino acids were missing from the TDRD6 mutant protein, resulting in significant changes in its 3D structure (Figure 1F). In addition, recombinant plasmids carrying human WT *TDRD6* and *TDRD6* c.G1825T cDNAs were constructed and transfected into HEK293T cells. The CHX chase experiments showed that the degradation stability of the mutant TDRD6 protein was significantly reduced (Figure S4).

### 3.2 Generation of *Tdrd6*‐mutant mice in which spermatogenesis was blocked at the round spermatid stage

**FIGURE 2:**
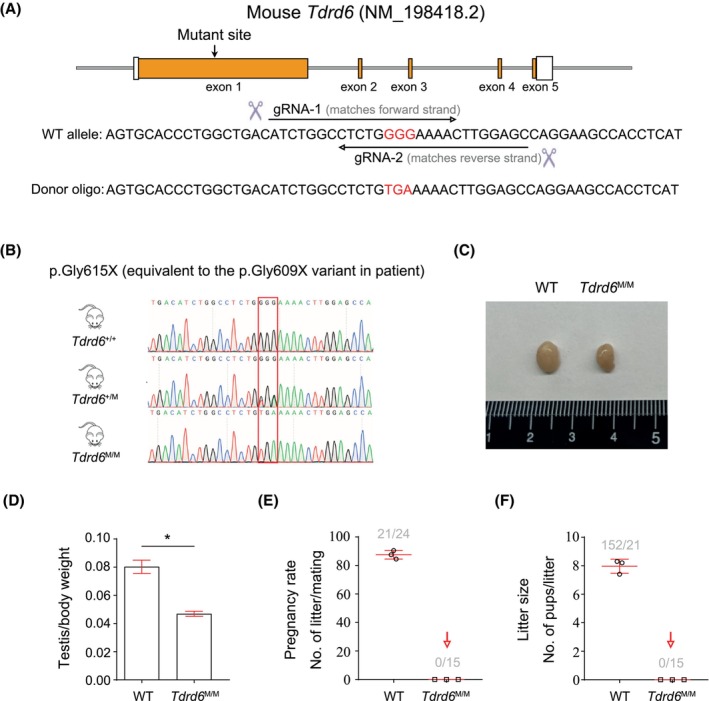
Generation of *Tdrd6*‐mutant mice. (A) Mice carrying a homozygous variant p.Gly615X in the *Tdrd6* gene equivalent to the p.Gly609X variant identified in the patient were generated using CRISPR/Cas9 technology. (B) Genotyping was examined by Sanger sequencing. GGG was edited to TGA (a stop codon). (C, D) Morphology of the testis and the testis/body weight ratio of *Tdrd6* ^M/M^ mice and WT mice. (E, F) Fertility test of three *Tdrd6* ^M/M^ male mice and three littermate WT male mice for 2 months. Each male mouse was mated with two WT female mice. *p < 0.05.

TDRD6 is a large protein (>200 kDa) with eight tudor repeats. It is not known how the lack of 4‐8 tudor domains affects the functions of TDRD6. The amino acid sequence of human TDRD6 is highly homologous to that of mouse (Figure S3B). Accordingly, we generated *Tdrd6*‐mutant mice. The p.Gly615X (GGG–TGA) variant that was created in mouse *Tdrd6* is equivalent to the p.Gly609X (GGA–TGA) variant of *TDRD6* identified in the patient (Figure 2A). Details of the construction and generation of the *Tdrd6*‐mutant mice are provided in Figure S1.

**FIGURE 3:**
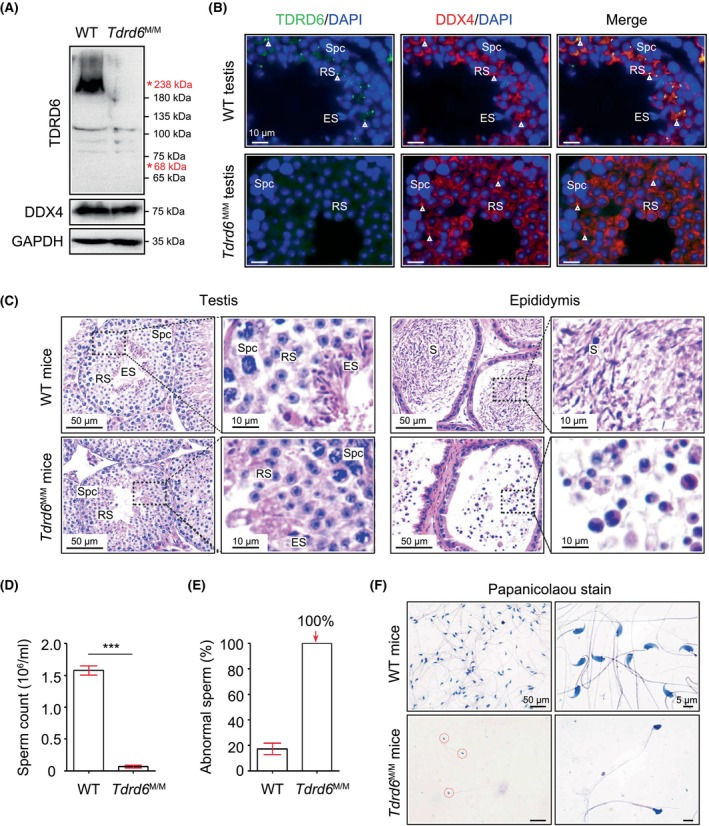
Round spermatid arrest in *Tdrd6* ^M/M^ mice. (A) Western blotting analysis of TDRD6 and DDX4 expression in the testis protein lysates of WT and *Tdrd6* ^M/M^ mice. GAPDH served as a loading control. TDRD6 is approximately 238 kDa, and the truncated TDRD6 (p.Gly615X) is expected to be approximately 68 kDa (indicated by red stars). (B) Double immunofluorescence staining of TDRD6 and DDX4 using testis sections from WT and *Tdrd6* ^M/M^ mice. ES, elongated spermatids; RS, round spermatids; Spc, spermatocytes. Arrowheads indicate the CBs in round spermatids. Scale bars, 10 μm. (C) Sections of testes and caudal epididymis were subjected to haematoxylin–eosin staining. Scale bars, 50 and 10 μm. (D) Sperm count (*n* = 3 in each group). Data are presented as mean ± SEM. Student's *t*‐test, ***p < 0.001. (E) Percentage of abnormal sperm (%) in *Tdrd6* ^M/M^ mice and their littermate WT mice. (F) Papanicolaou staining of sperm from adult WT and *Tdrd6* ^M/M^ mice. Scale bars, 50 and 5 μm.

The genotypes of *Tdrd6* ^+/+^, *Tdrd6* ^+/M^ and *Tdrd6* ^M/M^ were examined by PCR‐Sanger sequencing (Figure 2B). The testis size and weight of *Tdrd6* ^M/M^ mice were lower than those of WT mice (Figure 2C,D). The *Tdrd6* ^M/M^ mice were viable and showed no overt abnormalities; however, the fertility test showed that *Tdrd6* ^M/M^ male mice were completely infertile (Figure 2E,F). We generated antiserum of TDRD6 using recombinant proteins of TDRD6 (amino acids 300–608). *Tdrd6* ^M/M^ mice may generate a truncated TDRD6 protein of approximately 68 kDa (615 amino acids) or no TDRD6 protein due to the nonsense‐mediated mRNA decay mechanism. Western blotting analysis showed that no TDRD6 protein was detected in the testis protein lysates of *Tdrd6* ^M/M^ mice, whereas the abundance level of DDX4 (a CB component) was not altered (Figure 3A). Immunofluorescence staining further indicated that TDRD6 and DDX4 were colocalized in the CBs of round spermatids in WT mice (Figure 3B). In *Tdrd6* ^M/M^ mice, TDRD6 was absent in the testis section, whereas DDX4 was still localized in the CBs (and cytoplasm) of round spermatids (Figure 3B).

Histological analysis of adult testes showed that spermatogenesis was arrested at the round spermatid stage and some round germ cells were observed in the caudal epididymis of *Tdrd6* ^M/M^ mice (Figure 3C). The sperm count of *Tdrd6* ^M/M^ mice was significantly lower (almost none) than the sperm count of WT mice (Figure 3D). Papanicolaou staining showed that the very few remaining sperm in *Tdrd6* ^M/M^ mice had severe morphological abnormalities (Figure 3E,F). Taken together, our results show that *Tdrd6* ^M/M^ mice carrying p.Gly615X variant of *Tdrd6* (equivalent to the p.Gly609X variant of *TDRD6* identified in the patient) generated no TDRD6 protein and showed male infertility with round spermatid arrest, indicating this variant will completely disrupt the expression and function of TDRD6.

### 3.3 TDRD6 regulates the CB architecture by assembling of proteins into CBs

**FIGURE 4:**
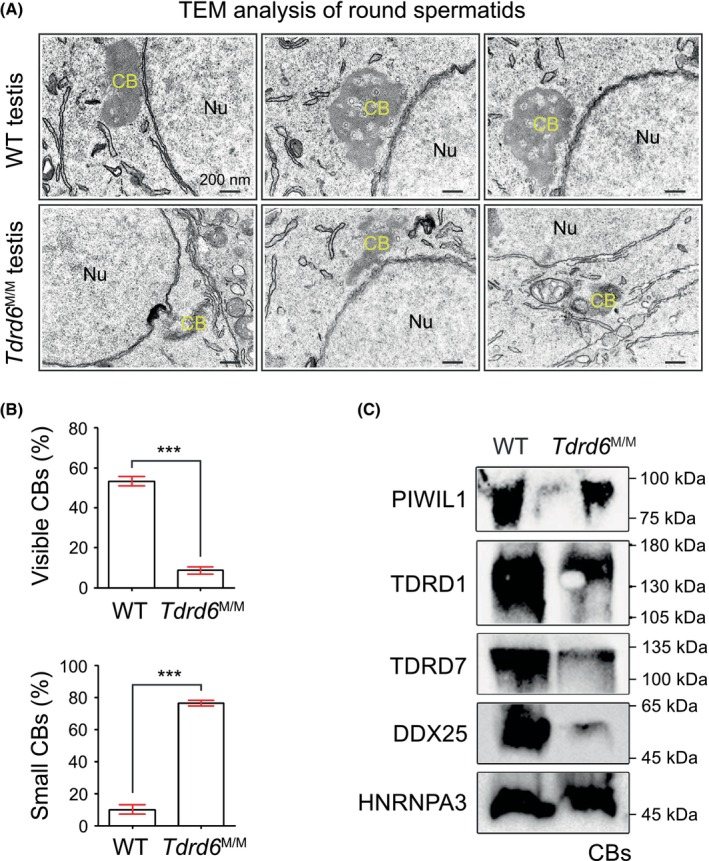
Disruption of CB architecture in *Tdrd6* ^M/M^ mice. (A) Transmission electron microscopy analysis of testes from WT and *Tdrd6* ^M/M^ mice. CBs were observed near the nuclear membrane in round spermatids (RS). Nu, nucleus. Scale bars, 200 nm. (B) Percentage of round spermatids with visible CBs (top) and percentage of small CBs (bottom) in WT and *Tdrd6* ^M/M^ mice (*n* = 3 in each group). Fifty round spermatids were counted in each group. Data are represented as mean ± SEM. Student's *t*‐test, ***p < 0.001. (C) PIWIL1, TDRD1, TDRD7, DDX25 and HNRNPA3 detected in CB samples from testicular cells of WT and *Tdrd6* ^M/M^ mice.

We further performed ultrastructural examination of CBs within mouse testes. Transmission electron microscopy (TEM) indicated that the CBs were cloud‐like structures in round spermatids of WT mice, whereas the CBs were either seldom observed or small in size in round spermatids of *Tdrd6* ^M/M^ mice (Figure 4A). The percentage of round spermatids with viable CBs was significantly reduced in *Tdrd6* ^M/M^ mice (Figure 4B), and the ratio of small CBs was significantly higher in *Tdrd6* ^M/M^ mice than it was in WT mice (Figure 4B). These observations favour the idea that TDRD6 is essential for the correct architecture of CBs in round spermatids.

Fanourgakis *et al*. isolated CBs from adult WT and *Tdrd6*‐KO testes for MS analysis.^24^ There were 158 proteins enriched exclusively in WT CB samples, indicating their assembly into CBs was dependent on TDRD6.^24^ Among the 158 proteins, four have been reported to be critical for CB architecture, including PIWIL1,^25^ TDRD1,^26^ TDRD7^17^ and DDX25.^27^ We purified CBs from *Tdrd6* ^M/M^ and WT mice and detected the expression of PIWIL1, TDRD1, TDRD7 and DDX25 (Figure 4C). Their expression was obviously reduced in the CB samples of *Tdrd6* ^M/M^ mice as compared with that of WT mice. HNRNPA3, a negative control, was present equally in CBs of *Tdrd6* ^M/M^ and WT mice (Figure 4C).

### 3.4 Exploration of the interactome of TDRD6 in CBs by IP‐MS

**FIGURE 5:**
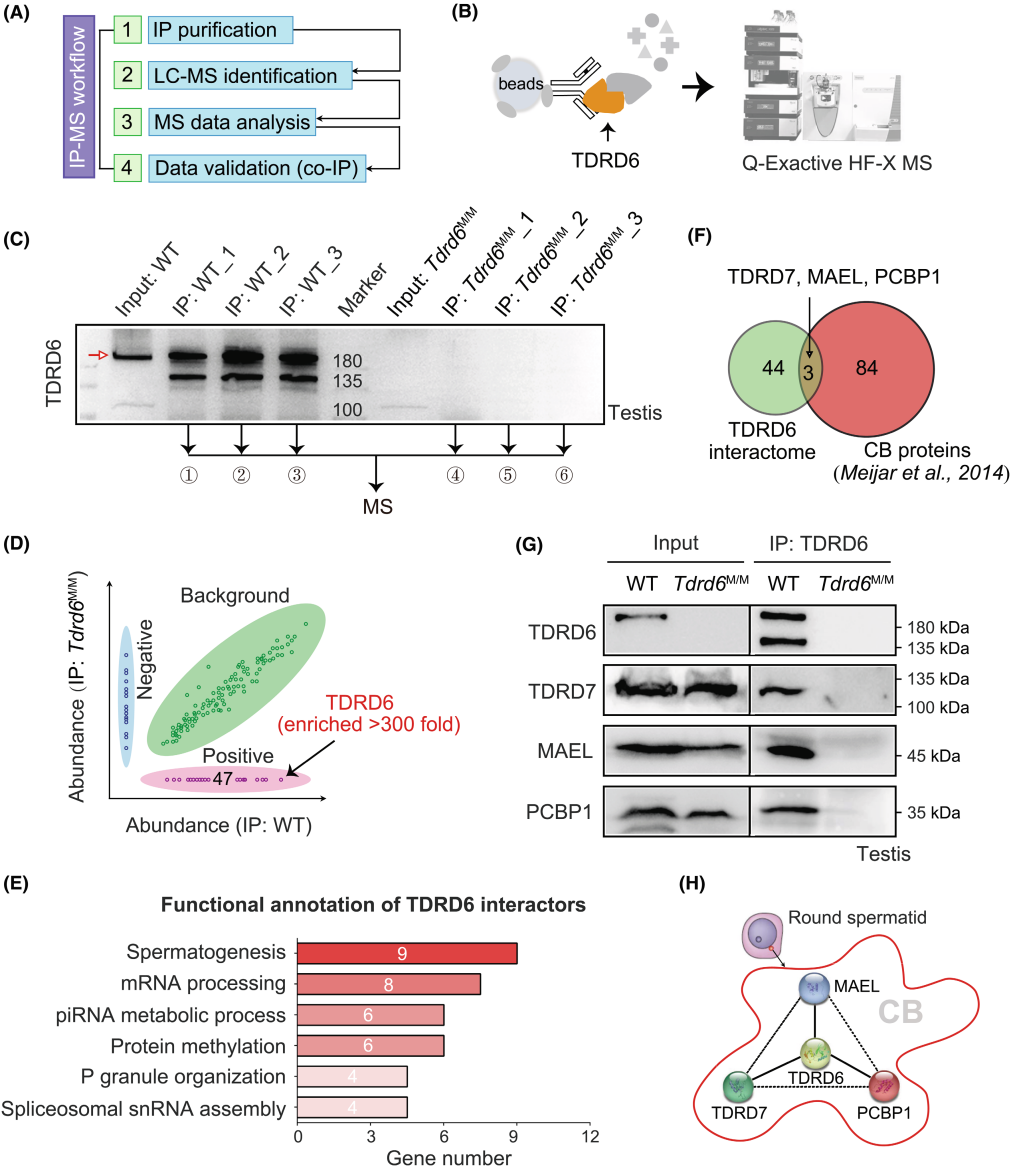
Interactome of TDRD6 in mouse testis protein lysates. (A) IP–MS workflow. (B, C) Endogenous TDRD6 protein and its interacting proteins were immunoprecipitated from testis protein lysates from 28‐day‐old WT and *Tdrd6* ^M/M^ mice (*n* = 3 in each group) by anti‐TDRD6 antiserum. The ‘Input’ group shows only one band (~230 kDa, indicated by the red arrow). The ‘IP’ groups have an extra band (135–180 kDa), which may be a potential isoform of TDRD6 or an unspecific band. (D) Forty‐seven differentially expressed proteins that were more enriched (fold change >4) in the WT‐IP group than they were in the *Tdrd6* ^M/M^‐IP group. (E) Functional annotation of TDRD6 interactors by gene ontology analysis. (F) Cross‐analysis was performed by using our TDRD6 interactome and a previously reported CB proteome. (G) The interaction between TDRD6 and TDRD7, MAEL or PCBP1 was confirmed by co‐IP experiments using mouse testis protein lysates and anti‐TDRD6 antiserum. (H) TDRD6 interacts with TDRD7, MAEL and PCBP1 within CBs.

Previous co‐IP studies suggested that TDRD6 interacted with several other components of CBs, including DDX4, PIWIL1, PIWIL2, UPF1 and UPF2.^16^, ^24^ To thoroughly explore the interactome of TDRD6, we performed IP‐MS of testis protein extracts from 28‐day‐old WT and *Tdrd6* ^M/M^ mice (*n* = 3 samples in each group) by using the specific TDRD6 antiserum (Figure 5A–C). Twenty‐eight‐day‐old mice were used because at this time the first wave of meiotic division occurred and round spermatids reached the peak of quantity. We identified 47 differentially expressed proteins that were enriched (fold change >4, in three independent replicates) in the WT‐IP group compared with those in the *Tdrd6* ^M/M^‐IP group (Figure 5D). TDRD6 was enriched >300‐fold in the WT‐IP group. All TDRD6‐interacting partners were listed in Table S3. TDRD6‐interacting proteins were involved mainly in spermatogenesis, mRNA processing, piRNA metabolic process, protein methylation, P granule organization and spliceosomal snRNA assembly pathways by gene ontology analysis (Figure 5E). The interaction between TDRD6 and three well‐known CB proteins (TDRD7, MAEL and PCBP1)^23^ was further confirmed by co‐IP assays using mouse testis protein lysates and their specific antibodies (Figure 5F–H).

### 3.5 ICSI outcome for the patient with the LOF *TDRD6* variant

Intracytoplasmic sperm injection (ICSI) is currently the most commonly used assisted reproductive technology (ART) for infertile men with OAT.^28^ The couple had undergone one ICSI cycle. In this attempt, only five MII oocytes were retrieved for ICSI, and a 20% fertilization rate was observed. However, the patient's wife was ultimately unable to undergo embryo transfer because of poor quality of the embryo (Table 3). Previous studies have demonstrated that abnormal localization and expression of the sperm‐specific protein PLCζ failed to trigger calcium oscillations in oocytes, leading to fertilization failure after ICSI.^29^, ^30^ Due to the poor fertilization of the proband and the unavailability of patient samples, we investigated the localization and expression of PLCζ in mice by immunofluorescence staining. As shown in Figure S5, the localization of PLCζ in *Tdrd6* ^M/M^ mice was altered, and the expression level of PLCζ was decreased. At the time of submission, the patient had not yet experienced a second ICSI cycle. Guo *et al*. showed that patients who carry bi‐allelic *TDRD6* variants have significantly reduced levels of PLCζ.^31^ Considering that artificial oocyte activation (AOA) is commonly used to improve fertilization rate following fertilization failure after ICSI,^32^ we will analyse PLCζ levels of the patient and consider using AOA in the subsequent ICSI cycles.

**TABLE 3:** Clinical outcomes of ICSI cycles using spermatozoa from man harbouring *TDRD6* variant.

	II‐1
Male age (years)	31
Female age (years)	29
Infertility years	7
Number of ICSI cycles	1
Number of oocytes injected	5
Number (and rate) of fertilized oocytes	1 (20%)
Number (and rate) of good quality embryos	0 (0%)
Number of transfer cycles	0
Number of embryos transferred per cycle	0
Implantation rate	/
Clinical pregnancy rate	/
Live birth rate	/

## 4 DISCUSSION

CBs are suggested to promote the compartmentalization and coordination of RNA regulatory pathways in male germ cells.^33^ Studies on CBs are of great significance for understanding the regulatory mechanism of spermatogenesis and the aetiology of male infertility. Previous studies have identified hundreds of CB components^23^ and shown that defects in CBs cause infertility in mice. For example, *Miwi*‐KO mice exhibit male sterility with arrested spermatogenesis at the round spermatid stage, and CB structure changes from compact to diffuse.^34^ Loss of *Tdrd5* in mice led to blocked spermatogenesis, disordered CB structure and mislocalization of other CB components.^35^

TDRD proteins are important CB components that recognize the N‐terminal arginine‐rich motif of PIWI proteins via their conserved extended tudor domain, which is essential in germ cell development.^13^ Clinical and animal evidences have highlighted the physiological role of TDRD family members, such as TDRD6, TDRD7 and TDRD9, in human and mouse male infertility (summarized in Table S4).^14^, ^15^, ^16^, ^17^, ^18^, ^19^ TDRD6 is a well‐known protein that is specifically expressed in CBs of round spermatids.^36^ *Tdrd6*‐KO male mice are infertile due to the blockade of spermatogenesis at the round spermatid stage.^16^ With the removal of TDRD6, CBs distort to a diffuse, less coagulated and ruptured appearance, accompanied by mislocalization of key components of CBs, including MVH, MIWI and MAEL.^16^ *TDRD6* variants have rarely been reported in humans. Only two previous studies identified bi‐allelic *TDRD6* variants in patients with OAT.^14^, ^31^

We performed the first interactome study of TDRD6 in mouse testes and identified 47 TDRD6‐interacting proteins. The interaction between TDRD6 and TDRD7, MAEL or PCBP1 were validated by co‐IP experiments. LOF *TDRD7* variants were found to lead to congenital cataract and NOA in humans.^15^ TDRD7 is essential for dynamic ribonucleoprotein remodelling of CBs, and male *Tdrd7*‐KO mice are sterile, with spermatogenesis arrested at the round spermatid stage.^17^ MAEL is indispensable for silencing transposon repression, piRNA levels and translation of spermiogenic mRNAs. *Mael*‐KO mice exhibit male sterility with meiotic failure or spermiogenic arrest, depending on the genetic background.^37^, ^38^ LOF variants in the *MAEL* gene have not been reported in patients with NOA or OAT. *Pcbp1*‐KO embryos lose viability at the blastocyst stage, and PCBP1 is involved in alternative splicing, microRNA processing, translation and RNA stability.^39^, ^40^, ^41^ However, the physiological role and mechanism underlying the regulation of CBs by PCBP1 are still unknown. Our interactome data provide a valuable source to study TDRD6‐interacting partners. As indicated by previous studies, TDRD6 interacts with MILI, MIWI, MVH and UPF1 by co‐IP experiments using protein lysates of cell lines and/or testes.^16^, ^24^ Surprisingly, these TDRD6‐interacting partners were not detected in our IP‐MS study, possibly because of the different methods used and efficiencies in the experiments. We are not suggesting that TDRD6 does not interact with these identified proteins, but that TDRD6 also interacts with other proteins, including TDRD7, MAEL and PCBP1.

For men with severe OAT, ICSI is an effective clinical treatment option that involves the direct injection of a single live spermatozoon into an oocyte to fertilize it.^42^ However, ICSI using spermatozoa from patients with severe OAT remains a challenge.^43^ Sha *et al*. performed ICSI for a patient harbouring a homozygous missense variant in *TDRD6* with a poor outcome. The patient's wife underwent two cycles of ICSI with separate embryo transfers, but was not pregnant after transplantation.^14^ Guo *et al*. reported that four patients with OAT who carried *TDRD6* variants and their wives were willing to receive ICSI treatment, but all experienced failed ICSI outcomes.^31^ In this study, the couple were treated with ICSI. However, only a 20% fertilization rate was observed, and no cleaved embryos were obtained.

In summary, we identified a novel homozygous LOF variant c.G1825T/p.Gly609X in *TDRD6* in an infertile man with severe OAT. *Tdrd6* ^M/M^ mice carrying the p.Gly615X variant (equivalent to the p.Gly609X variant in the patient) generated no TDRD6 protein (rather than a truncated protein) and showed severe OAT phenotype. Similar to *Tdrd6*‐KO mice, round spermatid arrest was observed in *Tdrd6* ^M/M^ mice. Loss of TDRD6 affected the CB assembly of PIWIL1, TDRD1, TDRD7 and DDX25; their knockout mice all shows a disruption of CB architecture. Furthermore, the TDRD6‐TDRD7‐MAEL‐PCBP1 complex was identified in CBs by applying IP‐MS and co‐IP. We also reported the ART outcomes of the patient who carried a homozygous LOF *TDRD6* variant. Our findings expand the mutational spectrum of severe OAT and provide new knowledge for the diagnosis and treatment of infertile men harbouring LOF *TDRD6* variants.

## AUTHOR CONTRIBUTIONS

**Xinying Bi:** Data curation (equal); writing – original draft (lead). **Huijuan Jin:** Formal analysis (equal). **Feng Wan:** Resources (equal). **Yanqing Xia:** Investigation (equal). **Haibin Guo:** Validation (equal). **Suren Chen:** Writing – review and editing (equal). **Binbin Wang:** Supervision (equal).

## FUNDING INFORMATION

This study was supported by the National Natural Science Foundation of China (32370905 to S.C.), Central Government to Guide Local Scientific and Technological Development (ZY21195023 to B.W.), Fundamental Research Funds for the Central Institutes (2023GJZD01 to B.W.), National Key Research and Development Project (2019YFA0802101 to S.C.), Open Fund of Key Laboratory of Cell Proliferation and Regulation Biology, Ministry of Education (to S.C.).

## CONFLICT OF INTEREST STATEMENT

The authors declare that they have no competing interests.

## Supporting information


Data S1:

Table S3.

## Data Availability

The mass spectrometry proteomics data have been deposited to the ProteomeXchange Consortium with the dataset identifier PXD042162. All data used during the study are available from the corresponding author on reasonable request.
