# Supplementary material for: Loss‐of‐function variant in TDRD6 cause male infertility with severe oligo‐astheno‐teratozoospermia in human and mice
Source: J Cell Mol Med. 2024 Sep 27;28(18):e18580. doi: 10.1111/jcmm.18580 (PMC11431060; doi:10.1111/jcmm.18580)
Supplement: Supplementary file 1 — Data S1: [file JCMM-28-e18580-s001.docx]

**Supplementary materials**

**
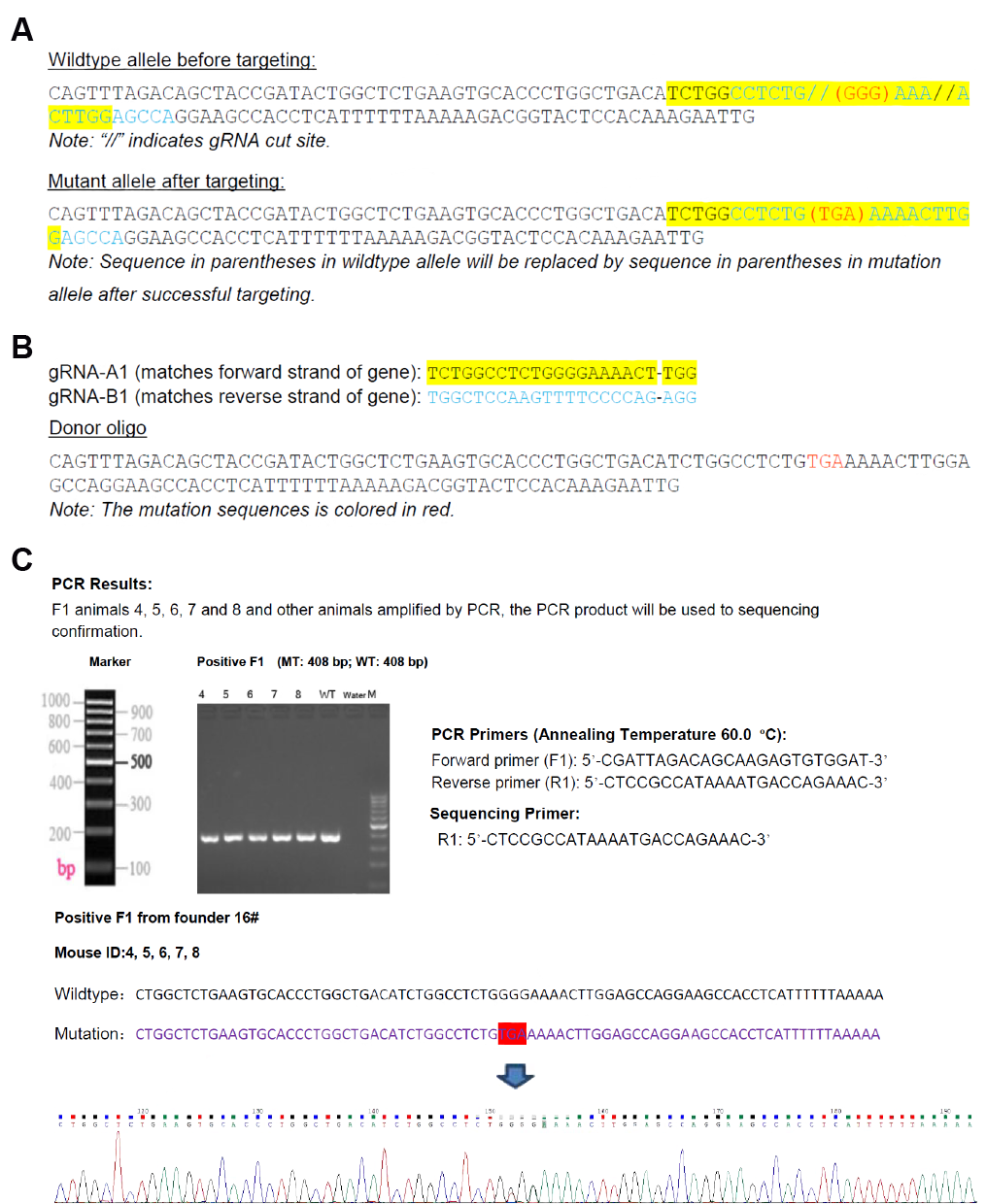
**

**Fig. S1. Animal report of mouse Tdrd6 (p.Gly615X) point mutation.**

**(A)** Wild-type allele before targeting and mutant allele after targeting are shown. p.Gly615X (GGG to TGA) was created in the mouse *Tdrd6* gene. “//” indicates the gRNA cut site. **(B)** Two gRNAs (gRNA-A1 and gRNA-B1) were designed, and gRNA-B1 was finally used to generate the founder mouse used in this study. The p.Gly615X (GGG to TGA) in the donor oligo was introduced into exon 1 by homology-directed repair. Cas9, gRNA and donor oligo were co-injected into fertilized eggs for KI mouse production. The pups will be genotyped by PCR followed by sequence analysis. **(C)** F0 founder animals were identified by PCR followed by sequence analysis and were bred to wild-type mice to test germline transmission and F1 animal generation. Positive F1 animals 4, 5, 6, 7 and 8 with p.Gly615X (GGG to TGA) mutation were confirmed by PCR and sequencing.


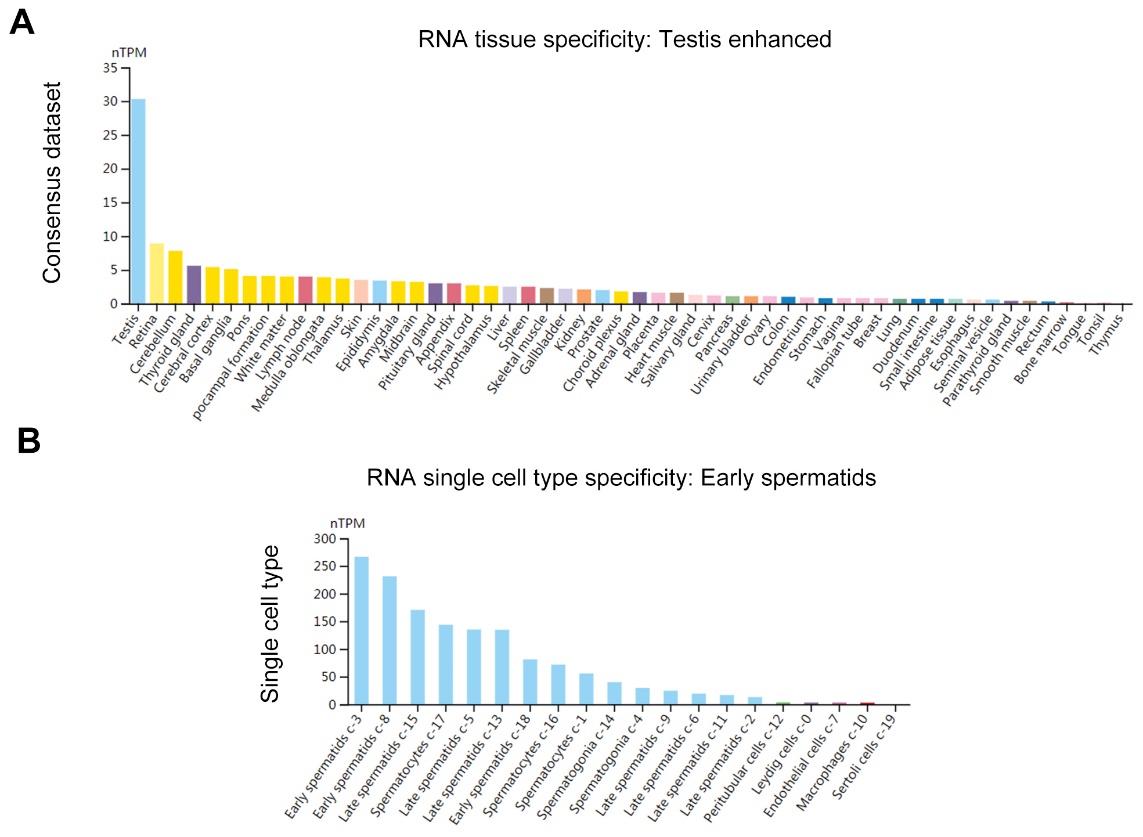


**Fig. S2. Expression information of human *TDRD6* mRNA.**

**(A)** The tissue data for mRNA expression was obtained from the Expression Atlas database (https://www.ebi.ac.uk/gxa). Human *TDRD6* mRNA was highly expressed in the testis tissue. **(B)** According to the Single Cell Expression dataset (https://www.ebi.ac.uk/gxa/sc), human *TDRD6* mRNA was restricted to early spermatids within the testes.

**
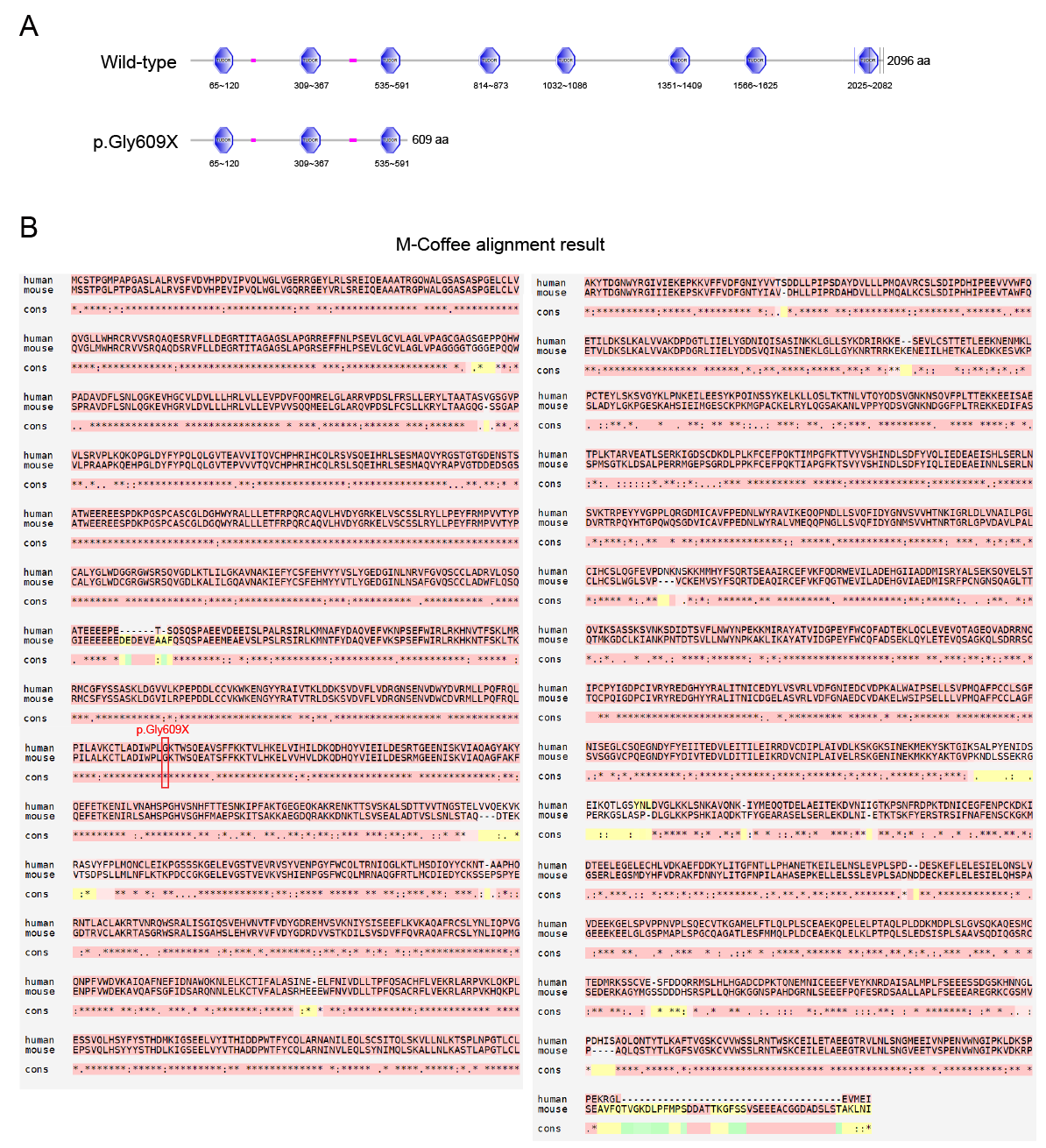
**

**Fig. S3. Alignment result of human and mouse TDRD6.**

**(A)** Wild-type human TDRD6 (~2096 amino acids) contains eight tudor domains. The p.Gly609X variant is expected to generate a truncated TDRD6 (~609 amino acids) lacking 4-8 tudor domains. **(B)** Alignment results of human and mouse TDRD6 were analysed by the M-Coffee website (<https://tcoffee.crg.eu/>). The p.Gly609X variant is indicated.

**
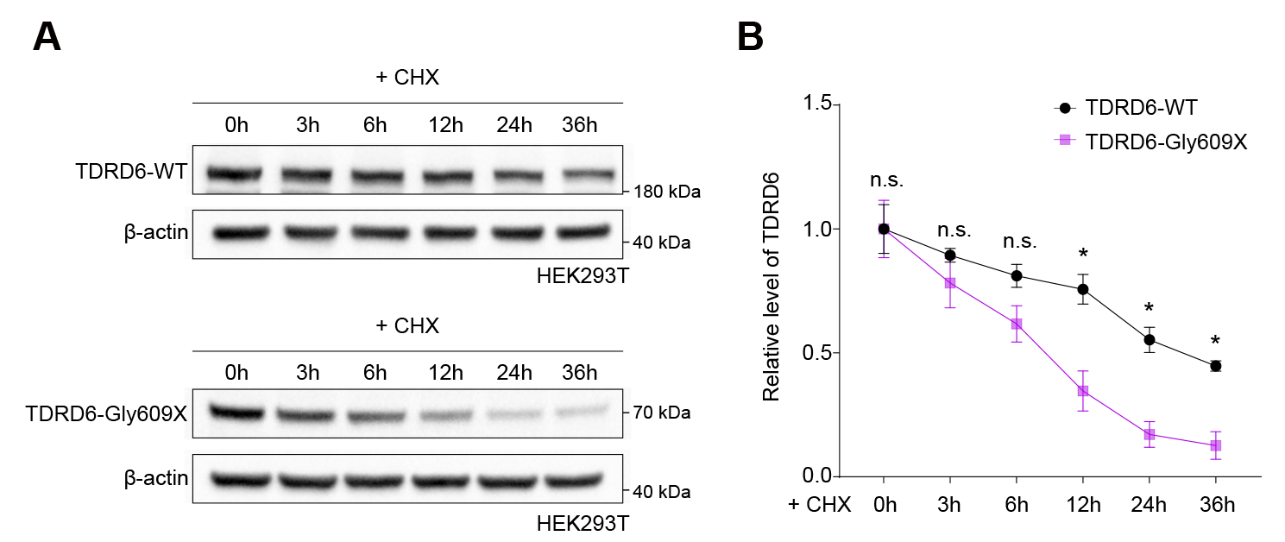
**

**Fig. S4. Comparison of TDRD6 stability between WT and p.Gly609X variant.**

**(A)** Representative Western blot analysis of TDRD6 expression after WT or mutant (c.G1825T/p.Gly609X) vector transfection for 48 hours in HEK293T cells followed by 100 μg/mL cycloheximide (CHX) treatment of the indicated time points. **(B)** Relative protein level of TDRD6 in the WT and mutant group at 0 h, 3 h, 6 h, 12 h, 24 h and 36 h after CHX treatment. Data were presented as the mean ± SEM (*n* = 3 each group) and band intensities were normalized to β-actin. Statistical significance was determined by two-tailed, unpaired Student's *t* test; **p*<0.05.

**
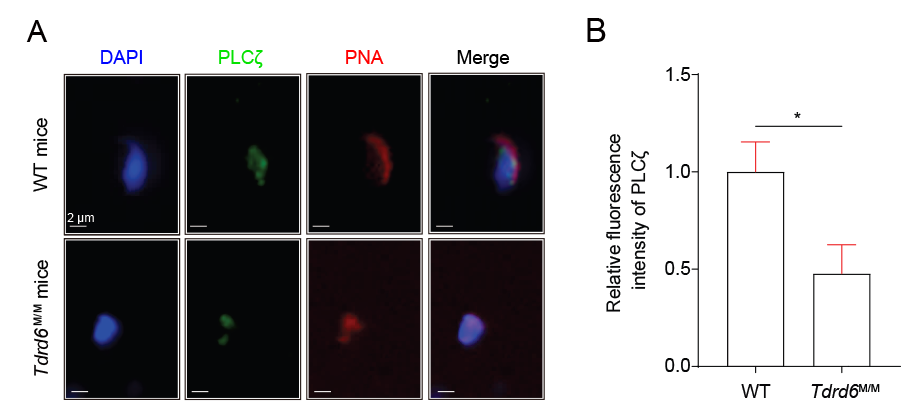
**

**Fig. S5. *Tdrd6* deficiency affects the localization and expression of PLCζ.**

**(A)** Immunofluorescence staining indicated abnormal PLCζ localization in sperm of *Tdrd6*^M/M^ mice. (Red: acrosome, green: PLCζ, blue: nuclei). **(B)** Statistical graph of the relative fluorescence intensity of PLCζ expression in sperm of WT and *Tdrd6*^M/M^ male mice. The experiments were independently performed at least three times. Data are represented as mean ± SEM. Student’s *t* test, **p*<0.05.

**Table S1. Primers for verification of *TDRD6* variant.**

| Primer name | Primer sequence (5’ to 3’) |
| --- | --- |
| *TDRD6*_variant Sanger-F | GATGAATGCCTTCTACGA |
| *TDRD6*_variant Sanger-R | TGCTCTGGACCACTGTCT |

**Table S2. All candidate variants identified in the patient by WES.**

| **Gene** | **Zygosity** | **Chr** | **Variant** | |
| --- | --- | --- | --- | --- |
| *AR* | HEM (for hemizygous) | X | NM_000044:exon1:c.1369_1383del:p.G469_G473del | |
| *ATRX* | HEM (for hemizygous) | X | NM_138270:exon8:c.G2671C:p.E891Q | |
| *EPHB6* | Hom | 7 | NM_001280795:exon4:c.G94T:p.A32S | |
| *ERICH2* | Hom | 2 | NM_001290030:exon1:c.T485G:p.L162R | |
| *GDF5* | Hom | 20 | NM_000557:exon2:c.G826T:p.A276S | |
| *IRS4* | HEM (for hemizygous) | X | NM_003604:exon1:c.T1534C:p.S512P | |
| *KDM5D* | HEM (for hemizygous) | Y | NM_001146705:exon5:c.A478G:p.I160V | |
| *OR4M2* | Hom | 15 | NM_001004719:exon1:c.G715A:p.V239M | |
| *PEX5* | Hom | 12 | NM_000319:exon1:c.136_147del:p.E48_S51del | |
| *PTCHD1* | HEM (for hemizygous) | X | NM_173495:exon1:c.C44G:p.S15C | |
| *RIMBP3B* | Hom | 22 | NM_001128633:exon1:c.T3184C:p.W1062R,RIMBP3B | |
| ***TDRD6*** | **Hom** | **6** | **NM_001010870:exon1:c.G1825T:p.G609X** | |
| *TMEM63B* | Hom | 6 | NM_001318792:exon4:c.G248A:p.R83Q | |
| *TPTE* | Hom | 21 | NM_001290224:exon14:c.A742G:p.K248E | |
| *ARID1B* | Het | 6 | NM_017519:exon17:c.G4309A:p.G1437S | |
|  | Het | 6 | NM_017519:exon17:c.T4681C:p.F1561L | |
| *CCL3L1* | Het | 17 | NM_001001437:exon3:c.T272C:p.L91P | |
|  | Het | 17 | NM_001001437:exon3:c.T193C:p.F65L | |
| *FMN1* | Het | 15 | NM_001103184:exon4:c.2103_2165del:p.701_722del | |
|  | Het | 15 | NM_001103184:exon4:c.C2092T:p.P698S | |
| *GPR142* | Het | 17 | NM_001331076:exon4:c.G752A:p.R251Q | |
|  | Het | 17 | NM_001331076:exon4:c.G854A:p.R285Q | |
| *MX1* | Het | 21 | NM_001282920:exon10:c.C1442G:p.P481R | |
|  | Het | 21 | NM_001282920:exon10:c.C1523T:p.P508L | |
| *WDR27* | Het | 6 | | NM_001350623:exon16:c.G1609C:p.E537Q |
|  | Het | 6 | | NM_001350625:exon13:c.G1246T:p.G416W |

**Table S4. Clinical and animal evidences highlight the role of TDRD family members in NOA or severe OAT.**

| **Gene** | **Variants** | **Patients’ Symptom** | **Phenotype of**  **KO/KI mice** |
| --- | --- | --- | --- |
| *TDRD6* | c.1259A>G/p.Y420C*^[1]^ | OAT^[1,2, this study]^ | Male sterility, round spermatid failure^[3, this study]^ |
|  | c.3275C>T/p.P1092L^[2]^ |  |  |
|  | c.1256A>G/p.Y419C^[2]^ |  |  |
|  | c.1563_1564del/p.C521Wfs*8^[2]^ |  |  |
|  | c.1834T>C/p.W612R^[2]^ |  |  |
|  | c.2690_2693del/p.V897Gfs*3^[2]^ |  |  |
|  | c.4019delG/p.S1340Mfs*10^[2]^ |  |  |
|  | c.3851C>G/p.S1284*^[2]^ |  |  |
|  | c.3217_3218dup/p.T1074*^[2]^ |  |  |
|  | c.G1825T/p.G609X^(this study)^ |  |  |
| *TDRD7* | c.324_325insA/T110Nfs*30^[4]^ | CC and NOA^[4]^ | Male sterility, round spermatid failure, CC^[4,5]^ |
|  | c.688_689insA/p.Y230X^[4]^ |  |  |
| *TDRD9* | c.720_723 del TAGT/p.S241Pfs*4^[6]^ | NOA^[6,7]^ | Male sterility, meiotic failure^[8]^ |
|  | c.3483_3484dup/p.S1162Ifs*3^[7]^ |  |  |

OAT, oligoasthenoteratozoospermia; CC, congenital cataract; NOA, nonobstructive azoospermia; * The pathogenicity of this point mutation is not known.

**References**

1. Sha YW, Wang X, Su ZY, Wang C, Ji ZY, Mei LB, Zhang L, Deng BB, Huang XJ, Yan W et al. TDRD6 is associated with oligoasthenoteratozoospermia by sequencing the patient from a consanguineous family. Gene. 2018;659:84-88.
2. Guo R, Wu H, Zhu XY, Wang GX, Hu KQ, Li KK, Geng H, Xu C, Zu CW, Gao Y, Tang DD, Cao YX, He XJ. Bi-allelic variants in chromatoid body protein TDRD6 cause spermiogenesis defects and severe oligoasthenoteratozoospermia in humans. J Med Genet. 2024;61(6):553-565.
3. Vasileva A, Tiedau D, Firooznia A, Müller-Reichert T, Jessberger R. Tdrd6 is required for spermiogenesis, chromatoid body architecture, and regulation of miRNA expression. Curr Biol. 2009;19(8):630-9.
4. Tan YQ, Tu CF, Meng LL, Yuan SM, Sjaarda C, Luo AX, Du J, Li W, Gong F, Zhong C et al. Loss-of-function mutations in TDRD7 lead to a rare novel syndrome combining congenital cataract and nonobstructive azoospermia in humans. Genet Med. 2019;21(5):1209-1217.
5. Tanaka T, Hosokawa M, Vagin VV, Reuter M, Hayashi E, Mochizuki AL, Kitamura K, Yamanaka H, Kondoh G, Okawa K et al. Tudor domain containing 7 (Tdrd7) is essential for dynamic ribonucleoprotein (RNP) remodeling of chromatoid bodies during spermatogenesis. Proc Natl Acad Sci U S A. 2011;108(26):10579-84.
6. Arafat M, Har-Vardi I, Harlev A, Levitas E, Zeadna A, Abofoul-Azab M, Dyomin V, Sheffield VC, Lunenfeld E, Huleihel M et al. Mutation in TDRD9 causes non-obstructive azoospermia in infertile men. J Med Genet. 2017;54(9):633-639.
7. Kherraf ZE, Cazin C, Bouker A, Fourati Ben Mustapha S, Hennebicq S, Septier A, Coutton C, Raymond L, Nouchy M, Thierry-Mieg N et al. Whole-exome sequencing improves the diagnosis and care of men with non-obstructive azoospermia. Am J Hum Genet. 2022;109(3):508-517.
8. Shoji M, Tanaka T, Hosokawa M, Reuter M, Stark A, Kato Y, Kondoh G, Okawa K, Chujo T, Suzuki T et al. The TDRD9-MIWI2 complex is essential for piRNA-mediated retrotransposon silencing in the mouse male germline. Dev Cell. 2009;17(6):775-87.
